# Enhancing study quality assessment: an in-depth review of risk of bias tools for meta-analysis—a comprehensive guide for anesthesiologists

**DOI:** 10.1186/s44158-023-00129-z

**Published:** 2023-11-06

**Authors:** Alessandro De Cassai, Annalisa Boscolo, Francesco Zarantonello, Tommaso Pettenuzzo, Nicolò Sella, Federico Geraldini, Marina Munari, Paolo Navalesi

**Affiliations:** 1 Sant’Antonio Anesthesia and Intensive Care Unit, University Hospital of Padua, Padua, Italy; 2 Anesthesia and Intensive Care Unit, University Hospital of Padua, Padua, Italy; 3 Anesthesia and Intensive Care Unit, Department of Medicine—DIMED, University of Padua, Padua, Italy; 4 Department of Cardiac, Thoracic, Vascular Sciences and Public Health, University of PaduaUOC Anesthesia and Intensive Care Unit, University Hospital of Padua, Padua, Italy

**Keywords:** Statistical analysis, Meta-analysis, Bias

## Abstract

**Background:**

Yearly, a multitude of randomized controlled trials are published, overwhelming clinicians with conflicting information; this data saturation leads to confusion and hinders clinicians’ everyday decision-making. Hence, it is crucial to assess the quality and reliability of the evidence in order to consolidate it. Through this synthesis, clinicians can guarantee that their decisions are informed by solid evidence. Meta-analysis, a statistical technique, can effectively combine data from multiple studies to furnish accurate and dependable evidence for clinical practice and policy decisions. Nonetheless, the reliability of the obtained results depends on the use of high-quality evidence.

**Main body:**

Risk of bias is an assessment mandatory while performing a meta-analysis and is used to have an overview of the quality of the studies from which data are extracted. Several tools have been developed and are used to perform the risk of bias assessment. In this statistical round, we will provide an overview of the most used tools for both the randomized (Cochrane Risk of Bias 2 and Jadad) and the nonrandomized (Risk Of Bias In Non-randomized Studies and Newcastle–Ottawa Scale) clinical trials.

**Conclusion:**

We provided an overview of the most used risk of bias tools used in meta-analysis.

Each year, numerous randomized controlled trials are released, flooding clinicians with a wealth of information that can sometimes lead to conflicting results. This influx of data often causes confusion and hampers clinicians’ everyday decision-making process [1]. In light of this, it becomes imperative to synthesize the evidence by evaluating its quality and the reliability of the results. By doing so, we can ensure that clinicians can make well-informed decisions based on solid evidence [2].

Meta-analysis is a powerful statistical method used to synthesize data from multiple studies, providing a more robust and accurate estimation of the effect of an intervention or exposure and it is at the top of the hierarchy of scientific knowledge. The process of meta-analysis involves identifying and selecting relevant studies, extracting data, and combining and analyzing the data to obtain a summary effect size. However, the validity and reliability of the meta-analysis depend on the quality of the included studies.

The quality of a study can be affected by various factors such as the study design, conduct, and reporting. These factors may introduce systematic errors, leading to a biased estimation of the effect size. The lower the quality of included studies, the lower the precision of obtained meta-analytic results. Thus, it is essential to evaluate the risk of bias in individual studies included in a meta-analysis to ensure that the meta-analysis is based on high-quality evidence.

The risk of bias refers to the potential for systematic error in a study’s design, conduct, or reporting that may lead to biased results. The risk of bias can arise from several sources such as inadequate randomization, allocation concealment, blinding, selective reporting, and incomplete outcome data. Therefore, it is crucial to assess the risk of bias in individual studies to identify potential sources of bias and minimize their impact on the meta-analysis findings. In fact, if the risk of bias is high in the included studies, the meta-analysis results may be biased and should be interpreted with caution. Conversely, if the risk of bias is low, the meta-analysis results are likely to be more reliable and can be used to inform clinical practice and policy decisions.

Several tools have been developed over the years, and the same tools have been improved as Cochrane Risk of Bias (RoB) 2 [3] from the previous Cochrane RoB [4] or extended as Risk Of Bias In Non-randomized Studies (ROBINS) of Exposures [5] from the ROBINS of Interventions tool [6].

The risk of bias evaluation is usually done by independent reviewers, who use standardized tools to assess the risk of bias in individual studies. The use of standardized tools ensures that the risk of bias evaluation is consistent and reliable across studies.

Several tools are available for assessing the risk of bias in different types of studies included in a meta-analysis, even if the most used tools in reviews of health interventions are Cochrane RoB and Jadad Scale [7] for randomized controlled trials and Newcastle–Ottawa Scale (NOS) [8] and ROBINS for the nonrandomized ones.

In this statistical round, we will provide an overview of the main risk of bias tools used for both randomized and nonrandomized trials trying to highlight their differences, their point of strength, and their limitations.

## Cochrane risk of bias 2

The Cochrane RoB 2 tool [3] has been proposed quite recently but is already a widely used tool for assessing the risk of bias in randomized controlled trials included in a systematic review or meta-analysis. It was developed by the Cochrane Collaboration in response to feedback and criticisms of the previous Cochrane Risk of Bias tool. The RoB 2 tool assesses the risk of bias in five domains, including the randomization process, deviations from intended interventions, missing outcome data, measurement of the outcome, and selection of the reported result.

The randomization domain assesses the method used to generate the allocation sequence, allocation concealment, and whether blinding was maintained. The deviations from the intended intervention domain assess the extent to which participants and investigators adhered to the intended interventions. The missing outcome data domain assesses whether the amount and reasons for missing data were balanced across groups and whether the methods used to handle missing data were appropriate. The measurement of the outcome domain assesses the methods used to measure the outcomes and whether they were objective and reliable. Finally, the selection of the reported result domain assesses whether the reported results were free from selective reporting.

**Fig. 1:**
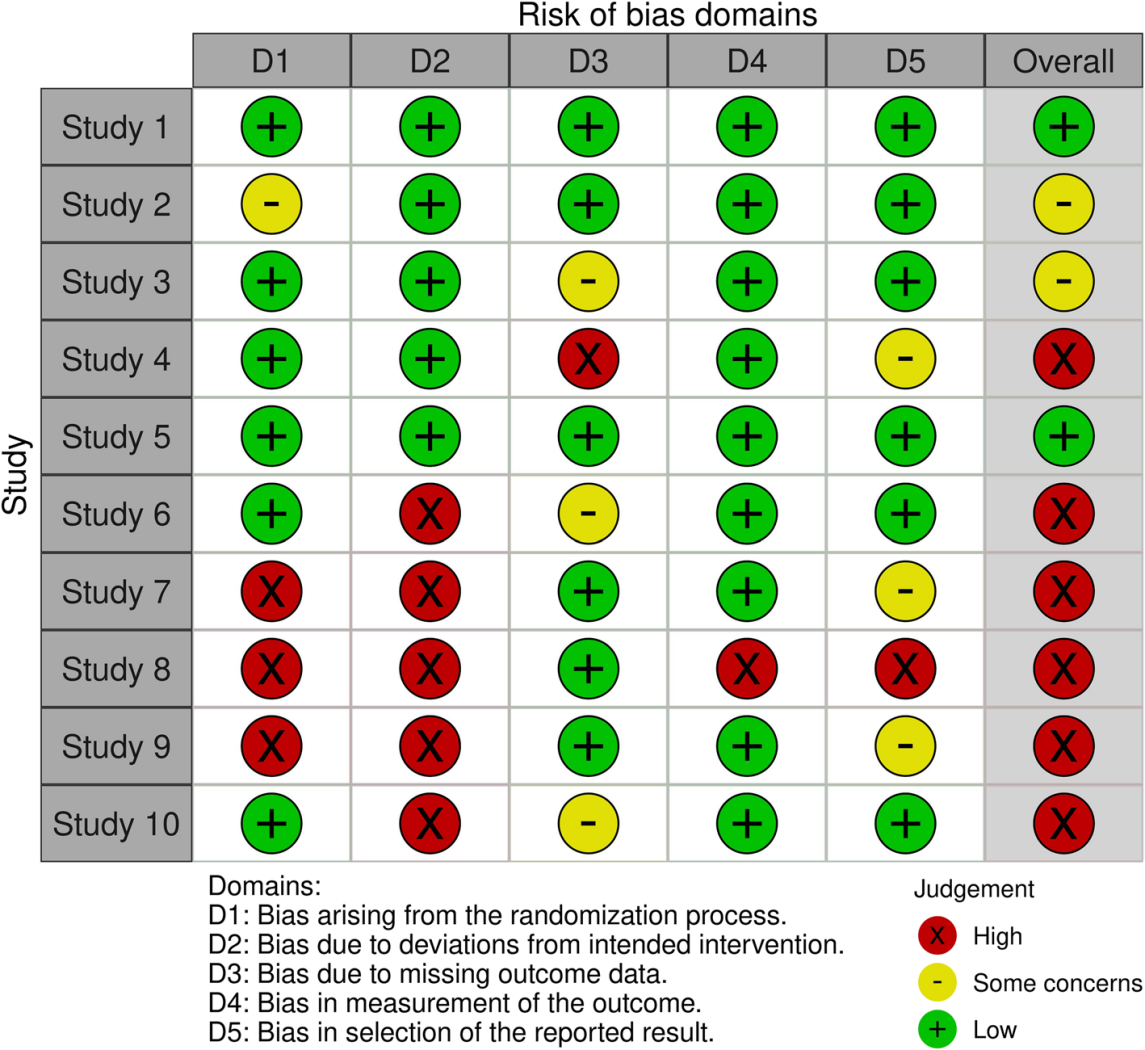
Example of Cochrane Risk of Bias 2 graphical representation

Each domain is evaluated using a set of signaling questions, which are used to judge the risk of bias as low, some concerns, or high. The overall risk of bias for each study is then rated as low, some concerns, or high based on the domain assessments, and usually, it is presented graphically with a red (high risk of bias), yellow (some concerns), and green (low risk of bias) image (Fig. 1).

Compared to the previous Cochrane Risk of Bias tool, the RoB 2 tool has several advantages. Firstly, the RoB 2 tool includes a more detailed assessment of the randomization process, including allocation concealment, which was not included in the previous tool. Secondly, the RoB 2 tool includes a domain to assess the risk of bias arising from deviations from intended interventions, which was not explicitly addressed in the previous tool. Thirdly, the RoB 2 tool includes a domain to assess the risk of bias arising from the selection of the reported result, which was not addressed in the previous tool. Finally, the RoB 2 tool provides clearer guidance on how to evaluate the risk of bias in each domain and how to rate the overall risk of bias. A study comparing the RoB 2 and the previous RoB has been performed [9]. The primary results suggest that the mean assessment times for both tools were similar for all outcomes, which was around 30 min, even though there was a learning curve associated with the new tool (RoB 2). The study reported that there were no significant usability issues with either tool, and they found few challenges with achieving agreement between raters. The primary difference between the two tools was with subjective outcomes in open-label studies, where RoB 1 was more likely to give harsher risk of bias assessments than RoB 2. The study concluded that RoB 2 was more beneficial than RoB 1 as it allowed reviewers to consider the results and endpoints in a better way, which improved the quality and relevance of risk of bias assessment. The authors also suggested some modifications to RoB 2 to improve its usability, such as changing the phrasing of certain signaling questions and implementing processes in the Excel tool to automatically populate study-based data that is not anticipated to vary across outcomes.

In conclusion, the Cochrane Risk of Bias 2 tool is a comprehensive and widely used tool for assessing the risk of bias in randomized controlled trials. It includes a more detailed assessment of the randomization process, deviations from intended interventions, missing outcome data, measurement of the outcome, and selection of the reported result compared to the previous Cochrane Risk of Bias tool. The RoB 2 tool provides clearer guidance on how to evaluate the risk of bias in each domain and how to rate the overall risk of bias, making it a valuable tool for researchers conducting systematic reviews and meta-analyses.

## Jadad scale

The Jadad Scale is a tool used to assess the methodological quality of clinical trials. It was developed in 1996 by Alejandro Jadad and colleagues [10] and is designed to assess the quality of randomized controlled trials.

The Jadad Scale consists of three items, each of which is scored on a scale to obtain a total score ranging from 0 to 5. The three items are:

1. Randomization: This item assesses the method used to generate the random sequence and whether allocation concealment was used to prevent selection bias. Studies that clearly describe a proper randomization method and allocation concealment receive a score of 2, while studies that do not meet these criteria receive a score of 0.
2. Blinding: This item assesses whether the study was double-blinded, meaning that both the participants and the investigators were blinded to the treatment assignment. Studies that report adequate blinding of both participants and investigators receive a score of 2, while studies that do not meet these criteria receive a score of 0.
3. Dropout and withdrawals: This item assesses the number of participants who dropped out or were lost to follow-up during the study and whether an intention-to-treat analysis was performed. Studies that report a low dropout rate and use an intention-to-treat analysis receive a score of 1, while studies that do not meet these criteria receive a score of 0.

As previously stated, the total possible score on the Jadad Scale is 5, with higher scores indicating higher methodological quality. Studies that score 3 or more are generally considered to be of high quality, while studies that score less than 3 are considered to be of low quality.

The Jadad Scale has several advantages as a tool for assessing the quality of randomized studies. Firstly, it is a simple and easy-to-use tool that can be quickly applied to a large number of studies. Secondly, it focuses on key methodological factors that are known to influence the validity of study results. Finally, it has been widely used and validated in a variety of settings, which provides some reassurance about its reliability and validity.

However, the Jadad Scale also has some limitations. It does not capture several aspects describing the quality of the study that have been, for example, formally incorporated into the CONSORT (Consolidated Standards of Reporting Trials) checklist [11]. For example, some of the chapters make reference to blinding of allocation, a priori sample size calculation, and statistical adjustment for multiple testing, particularly in areas that are not covered by the three items. Secondly, it may not be appropriate for all types of clinical trials, particularly those that use non-standard methodologies or interventions.

In conclusion, the Jadad Scale is a simple and widely used tool for assessing the quality of RCTs. It consists of three items that assess randomization, blinding, and dropout and withdrawals and is scored on a scale of 0 to 5. While the Jadad Scale has some limitations, it remains a useful tool for informing meta-analyses and systematic reviews and for identifying high-quality studies that can be used to inform clinical practice.

## Newcastle–Ottawa scale

The NOS [8] is a tool used to assess the risk of bias in non-randomized studies, such as cohort and case–control studies, that are included in a systematic review or meta-analysis. The NOS was first developed in the 1990s by researchers at the University of Newcastle and the Ottawa Hospital Research Institute and has since been widely used by researchers and reviewers to evaluate the quality of non-randomized studies.

The NOS assesses the risk of bias in three domains, including the selection of the study groups, the comparability of the groups, and the ascertainment of the outcome of interest. The selection domain assesses whether the study groups were selected in a way that minimized the risk of bias, such as whether the inclusion and exclusion criteria were clearly defined and whether the study groups were representative of the population of interest. The comparability domain assesses whether the study groups were similar with respect to important factors that could influence the outcome of interest, such as age, sex, and comorbidities. The ascertainment domain assesses whether the outcome of interest was ascertained in a way that minimized the risk of bias, such as whether the outcome was objectively measured or whether the outcome assessors were blinded to the exposure status.

Each domain is evaluated using a set of criteria, and each criterion is assigned a score of 0, 1, or 2, depending on the level of risk of bias. The total score for a study ranges from 0 to 9, with higher scores indicating a lower risk of bias. In addition, the NOS provides guidance on how to interpret the scores, such as categorizing studies with scores of 0–3, 4–6, and 7–9 as having high, moderate, or low risk of bias, respectively.

The NOS has several advantages as a tool for assessing the risk of bias in non-randomized studies. Firstly, it is a relatively simple and straightforward tool that can be applied to a wide range of non-randomized study designs. Secondly, it provides clear guidance on how to evaluate the risk of bias in each domain, which can help to ensure consistency across studies. Thirdly, it has been widely used and validated in a variety of settings, which provides some reassurance about its reliability and validity.

However, the NOS also has some limitations; it does not explicitly address some important sources of bias, such as confounding and selective reporting and it does not provide a single summary score of the overall risk of bias, which can make it difficult to compare the quality of different studies.

## ROBINS-I

The ROBINS-I tool [6] is a comprehensive instrument designed to assess the risk of bias in non-randomized studies of interventions, including observational studies, quasi-experimental designs, and other types of non-randomized study designs. The ROBINS-I tool was developed by an international group of experts in systematic reviews and meta-analyses, with the aim of improving the accuracy and consistency of risk of bias assessments for non-randomized studies.

The ROBINS-I tool assesses the risk of bias in seven domains, including confounding, selection of participants, classification of interventions, deviations from intended interventions, missing data, measurement of outcomes, and selection of reported results. Each domain is evaluated using a set of criteria, and each criterion is assigned a score of “low,” “moderate,” “serious,” “critical,” or “no information,” depending on the level of risk of bias.

The confounding domain assesses the extent to which the study design has controlled for potential confounding variables, such as age, sex, and comorbidities, that may be related to both the exposure and outcome of interest. The selection of participants’ domain assesses the extent to which the study sample is representative of the target population and the extent to which selection bias has been minimized. The classification of interventions’ domain assesses the extent to which the intervention or exposure of interest has been accurately and consistently defined and measured.

**Fig. 2:**
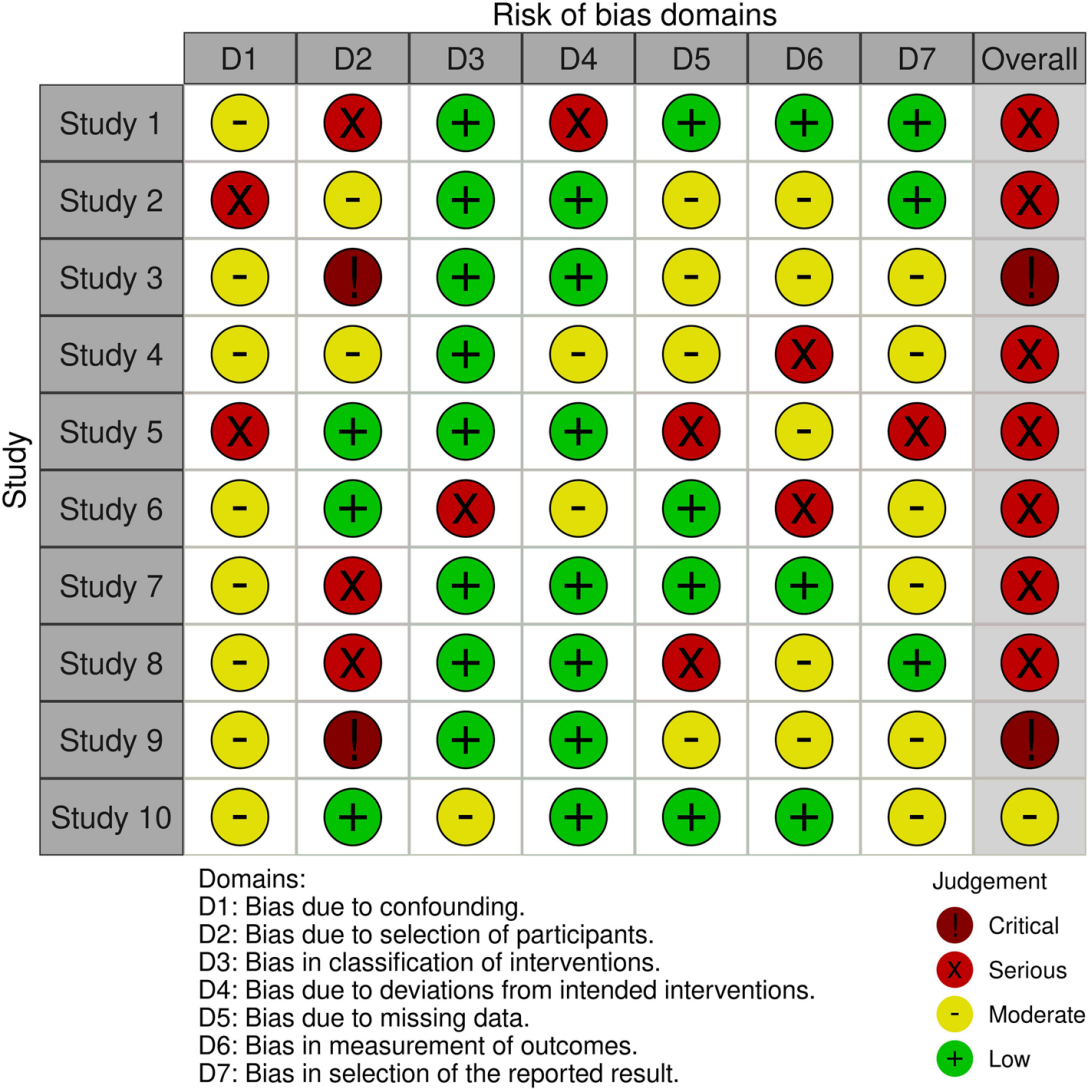
Example of Robins-I graphical representation

The deviations from the intended interventions domain assess the extent to which the study participants actually received the intervention or exposure of interest, as intended by the study protocol. The missing data domain assesses the extent to which missing data may have biased the study results, and whether the missing data have been addressed appropriately. The measurement of outcomes domain assesses the extent to which the outcome measures are valid, reliable, and unbiased. Finally, the selection of the reported results domain assesses the extent to which the study results are complete, accurate, and unbiased. The graphical output of ROBINS-I is depicted in Fig. 2.

Each study is assigned an overall risk of bias rating based on the highest risk of bias score across all domains. The possible overall ratings are “low,” “moderate,” “serious,” or “critical.” The ROBINS-I tool also provides guidance on how to interpret the ratings, such as categorizing studies with “low” or “moderate” risk of bias as having some confidence in the results and studies with “serious” or “critical” risk of bias as having limited confidence in the results.

While the ROBINS-I tool is reliable and investigates several domains of nonrandomized trials, it may appear complex and time-consuming; moreover, it may not be appropriate for all types of non-randomized study designs.

## ROBINS-E

**Fig. 3:**
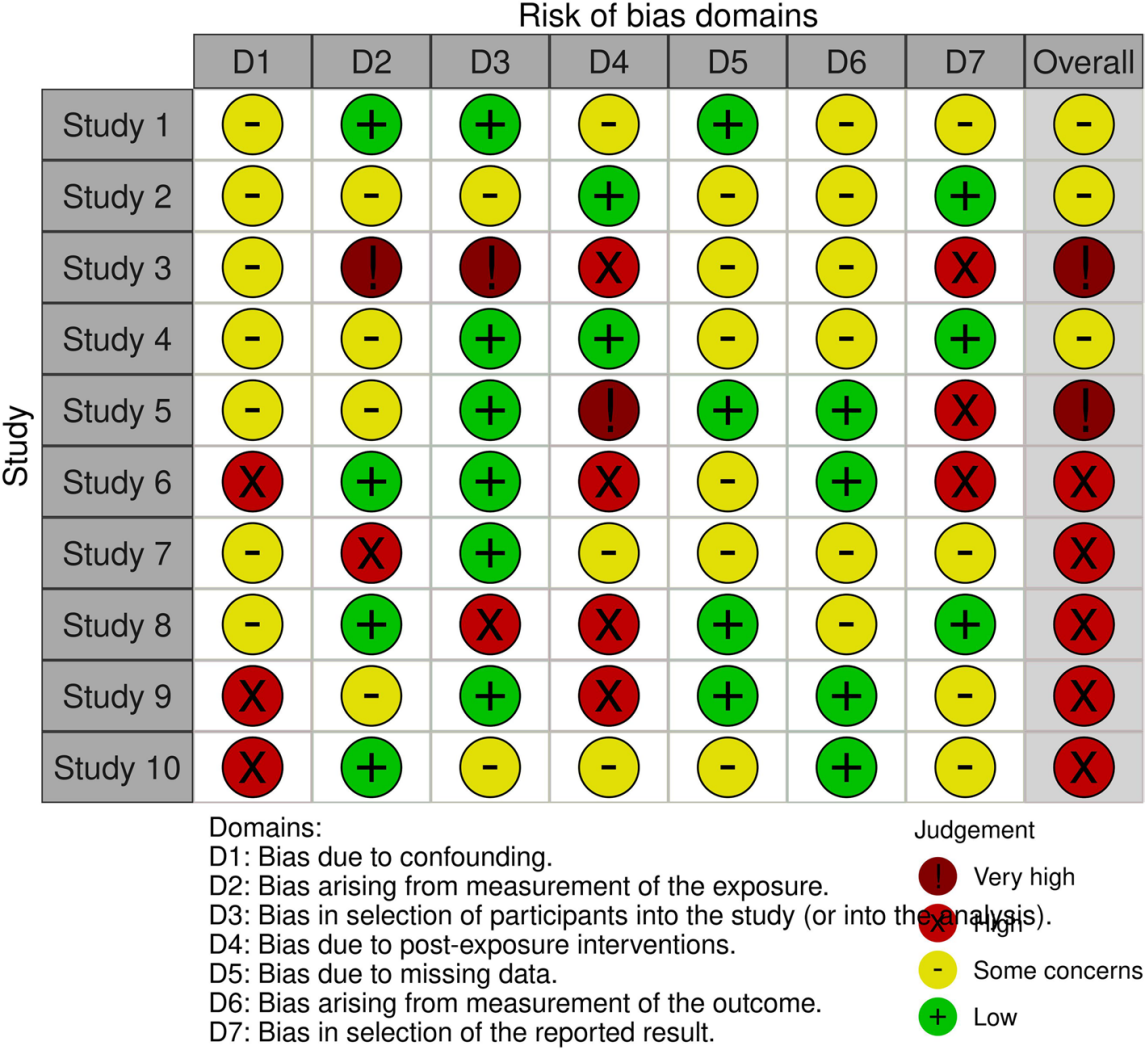
Example of Robins-E graphical representation

The ROBINS-E tool [5] is an extension of the ROBINS-I tool, which is used to assess the risk of bias in non-randomized studies. While the ROBINS-I tool assesses bias in non-randomized studies of interventions, the ROBINS-E tool is specifically designed for studies that investigate the effects of exposures. Like the ROBINS-I tool, the ROBINS-E tool includes assessments of bias due to confounding, selection bias, and measurement of outcomes, but also includes additional domains such as selection of the exposed and unexposed groups, measurement of exposure, and missing data. Overall, the ROBINS-E tool provides a more comprehensive assessment of the risk of bias in studies of exposures, while the ROBINS-I tool is designed for studies of interventions. The graphical output of ROBINS-E is then similar to ROBINS-I and an illustrative example is provided in Fig. 3.

## Selecting the appropriate tool for risk of bias assessment

Selecting the appropriate tool for assessing the risk of bias holds significant importance in the design of a meta-analysis and can greatly impact the resulting outcomes. In fact, comparative studies have demonstrated that different tools may lead to varying assignments of studies into high or low risk of bias categories which have important consequences on sensitivity analysis and on the overall confidence on the results, at the end of the day, only the best available evidence should inform clinical and policy decisions [12].

Compared to the Jadad scale, ROB2 provides a more conservative assessment of study quality, as it may assign a higher risk of bias scores even to studies with good Jadad scores [12]. This finding is to be expected given that ROB2 is a more comprehensive tool that requires more time for evaluation but critically analyzes additional domains, leading to a more thorough and detailed assessment of the studies.

Given the above, it comes as no surprise that ROB2 has emerged as the preferred tool in anesthesiology research, showing a consistent upward trend over the years. In contrast, the Jadad scale, which was commonly employed in the past, is now seldom seen in modern-day meta-analyses [13]. The authors recommend prioritizing the use of ROB2 for handling RCTs. However, if there are limited resources available to conduct thorough analyses, researchers may consider the Jadad scale.

An analogous argument can be made for non-randomized trials. Just like ROB2 for randomized controlled trials, the use of ROBINS-I for assessing non-randomized trials is also time-consuming and requires advanced knowledge in epidemiology [14]. It has been estimated that evaluating a single study using ROBINS-I can take anywhere between 3 and 7 h, whereas using NOS with a similar level of reliability only takes 30 min [15]. However, it is important to note that assessing the risk of bias using these two tools may lead to contradictory conclusions due to the reasons mentioned earlier [16]. On the same line, a study published in 2021 suggested that ROBINS-I is a more precise and rigorous tool than NOS; however, it also highlights that it is severely more time-consuming [17], While considering the non-randomized controlled trials, both ROBINS and NOS could be implemented and it is not mandatory to choose one over the other. However, the Cochrane Scientific Committee highly suggests using the ROBINS-I tool for the new reviews.

## Conclusions

While the risk of bias tools is valuable for assessing the quality of studies, they have limitations that researchers should consider when choosing the best tool. All the described tools are subjective and highly rely on the judgment of the reviewer, which can lead to variability in assessments. Another limitation is that the tools may not capture all aspects of study quality, and some domains may be more relevant than others depending on the research question and study design, while some tools investigate more domains and appear to be preferable to investigate the risk of bias they pose the risk to be more complex and time-consuming than others, which can make them impractical for use in some settings.

To overcome these constraints, it is important for researchers to take into account various aspects when selecting the most suitable tool. Before commencing the review, the research team should choose a relevant tool depending on the study design of the studies included, the most significant domain for that study design, and assess the practicability of adopting intricate and time-consuming tools in the research team setting. Moreover, familiarity, prior experience, and proficiency with the tools should also be considered when opting for a particular tool. By doing so, researchers can ensure an accurate assessment of study quality and improve the validity and reliability of their findings.

In conclusion, the evaluation of the risk of bias is a critical component of the systematic review process, and it is essential for the validity and reliability of the meta-analysis findings. The use of standardized tools and independent reviewers ensures consistency and reliability in the risk of bias evaluation. By identifying potential sources of bias, the risk of bias evaluation helps to minimize the impact of bias on the meta-analysis results, providing more accurate and reliable evidence for clinical practice and policy decisions.

## Data Availability

Not applicable.

## Abbreviations

NOS: Newcastle–Ottawa Scale
RoB: Cochrane Risk of Bias
ROBINS: Risk Of Bias In Non-randomized Studies
